# Do High Mental Demands at Work Protect Cognitive Health in Old Age *via* Hippocampal Volume? Results From a Community Sample

**DOI:** 10.3389/fnagi.2020.622321

**Published:** 2021-01-13

**Authors:** Francisca S. Rodriguez, Sebastian Huhn, William A. Vega, Maria P. Aranda, Matthias L. Schroeter, Christoph Engel, Ronny Baber, Ralph Burkhardt, Markus Löffler, Joachim Thiery, Arno Villringer, Tobias Luck, Steffi G. Riedel-Heller, A. Veronica Witte

**Affiliations:** ^1^German Center for Neurodegenerative Diseases (DZNE), RG Psychosocial Epidemiology and Public Health, Greifswald, Germany; ^2^Center for Cognitive Science, University of Kaiserslautern, Kaiserslautern, Germany; ^3^Institute of Social Medicine, Occupational Health and Public Health (ISAP), University of Leipzig, Leipzig, Germany; ^4^LIFE—Leipzig Research Center for Civilization Diseases, University of Leipzig, Leipzig, Germany; ^5^Max-Planck-Institute for Human Cognitive and Brain Sciences, Leipzig, Germany; ^6^Clinic for Cognitive Neurology, University Hospital Leipzig, Leipzig, Germany; ^7^Collaborative Research Centre 1052 “Obesity Mechanisms,” Subproject A1, Faculty of Medicine, University of Leipzig, Leipzig, Germany; ^8^Edward Royball Institute of Aging, University of Southern California, Los Angeles, CA, United States; ^9^Institute for Medical Informatics, Statistics and Epidemiology (IMISE), University of Leipzig, Leipzig, Germany; ^10^Institute of Laboratory Medicine, Clinical Chemistry and Molecular Diagnostics (ILM), University Hospital Leipzig, Leipzig, Germany; ^11^Faculty of Applied Social Sciences, University of Applied Sciences Erfurt, Erfurt, Germany

**Keywords:** hippocampus, cognitive functioning, mental demands, intellectual activities, aging

## Abstract

As higher mental demands at work are associated with lower dementia risk and a key symptom of dementia is hippocampal atrophy, the study aimed at investigating the association between mental demands at work and hippocampal volume. We analyzed data from the population-based LIFE-Adult-Study in Leipzig, Germany (*n* = 1,409, age 40–80). Hippocampal volumes were measured *via* three-dimensional Magnetic resonance imaging (MRI; 3D MP-RAGE) and mental demands at work were classified *via* the O*NET database. Linear regression analyses adjusted for gender, age, education, APOE e4-allele, hypertension, and diabetes revealed associations between higher demands in “language and knowledge,” “information processing,” and “creativity” at work on larger white and gray matter volume and better cognitive functioning with “creativity” having stronger effects for people not yet retired. Among retired individuals, higher demands in “pattern detection” were associated with larger white matter volume as well as larger hippocampal subfields CA2/CA3, suggesting a retention effect later in life. There were no other relevant associations with hippocampal volume. Our findings do not support the idea that mental demands at work protect cognitive health *via* hippocampal volume or brain volume. Further research may clarify through what mechanism mentally demanding activities influence specifically dementia pathology in the brain.

## Introduction

Mentally demanding activities at work seem to delay cognitive decline and lower dementia risk (Valenzuela and Sachdev, 2006a,b), as longitudinal studies (Smyth et al., 2004; Karp et al., 2009), as well as twin studies (Andel et al., 2005; Potter et al., 2007), have shown. Therein, it seems that the effect depends on the type of mental demand at work (MDW). A recent analysis of a prospective multi-center cohort study following 2,315 individuals up to 11 years has shown that higher MDW involving “information processing” (e.g., analyzing data) and “pattern detection” (e.g., detecting a figure, object, word, or sound that is hidden in other distracting material) relate to lower dementia risk in general, and high MDW involving “mathematics” and “creativity” delay dementia onset (Then et al., 2015). In that cohort, other MDW did not significantly affect dementia risk at all. To date, it is unclear why some demands may be more protective against dementia than others.

One possible explanation for this phenomenon might be related to potential mechanisms of how MDW and related factors alter dementia risk. However, these mechanisms, particularly at the neurobiological level, are largely unknown. Evidence on this subject matter is sparse. One study reported that, among young adults, the cognitive complexity of work demands is associated with better white matter integrity (Kaup et al., 2018). The only study with older adults, that we could identify, matched people by cognitive status and observed that job complexity was associated with a smaller hippocampal volume and more brain atrophy (Boots et al., 2015). Hippocampal volume declined with older age (Fjell et al., 2014; Fraser et al., 2015) and accelerated hippocampal atrophy is implicated in Alzheimer’s dementia (AD; Kaye et al., 1997; den Heijer et al., 2010). Hence, possibly, the hippocampus may play a role in the protective effects of mental demands at work on cognitive health. First human interventional studies implementing high-resolution magnetic resonance imaging (MRI) suggested that modifiable factors such as cognitive and physical activity exert protective effects on cognitive health *via* improvements in hippocampus plasticity (Duzel et al., 2016). For example, two months of memory training compared to placebo increased hippocampal volume in a group of older adults (Engvig et al., 2014), and AD patients with high educational attainment seem to have a larger hippocampal volume (Shpanskaya et al., 2014). Exposure to higher demands may thus also work on this pathway. However, the evidence is still sparse and it remains unclear whether long-lasting mental stimulation preserves hippocampus plasticity.

The study aimed to explore whether mental stimulation at work protects cognitive health by preserving hippocampal volume. Specifically, we investigated whether five types of MDW (language and knowledge, information processing, pattern recognition, mathematics, and creativity at work) were associated with hippocampal volume in a cross-sectional analysis of the large community-based “Adult Study” of the Leipzig Research Centre for Civilization Diseases (LIFE). As the effect may be dependent on being active in the workforce, we conducted the analyses separately for those working and those retired. Also, we tested whether hippocampal and brain volume (HBV) mediates the association between MDW and cognitive performance.

## Materials and Methods

### Study Design

We analyzed data of the “Adult Study” of the Leipzig Research Centre for Civilization DZNE (LIFE), a large population-based study investigating the prevalence, early onset markers, genetic predispositions, and the role of lifestyle factors in major civilization diseases. The details of the study have been described by Loeffler et al. (2015). Briefly, a random age- and a sex-stratified sample of residents of the city of Leipzig was obtained from the residents’ registry office. A letter of invitation to participate in the study was sent to every individual on the list. The only exclusion criterion was being pregnant.

The LIFE-Adult Study was conducted between August 2011 and November 2014. The study was approved by the ethics committee of the Medical Faculty of the University of Leipzig and was carried out in conformity with the principles embodied in the Declaration of Helsinki. All participants signed written informed consent before participation.

The assessments included physical and medical examinations, self-administered questionnaires, and psychometric testing, which were administered by trained study assistants and monitored by experienced scientists following standardized study protocols (Loeffler et al., 2015). A subsample of participants completed MRI at a second examination date (random sample from population registry, *n* = 2,637, 18–80 years). From these, we included all participants age 40–80 years (*n* = 2,271) from the analysis. Three-hundred and four participants were excluded due to major brain pathology (e.g., stroke, multiple sclerosis, tumors) or bad image quality (e.g., motion artifact). Another 10 participants were excluded because they reported having been diagnosed with a neurological or psychiatric disorder (*n* = 4 substance-related disorder, *n* = 2 human immunodeficiency virus (HIV), *n* = 2 Parkinson’s disease, *n* = 1 multiple sclerosis, *n* = 1 epilepsy). None of the participants included in the analyses had dementia or major neurocognitive disorder; we verified *via* cognitive testing (see “Cognitive Performance” section). We also excluded 74 participants because they were unemployed or retired with a total unemployment period of more than three years during their life. Further, individuals with missing data on important covariates were excluded: *n* = 128 missing data on apolipoprotein E (APOE) ε4 genotype, *n* = 26 missing data on having diabetes, *n* = 101 missing data on hypertension, and *n* = 117 occupational information could not be matched to O*NET database. And another *n* = 102 were excluded due to incomplete or invalid cognitive testing. The total number of participants in the analysis was n = *n* = 1,409.

### Hippocampal and Brain Volume

To estimate volumes, we used the three-dimensional Magnetization-Prepared Rapid Gradient Echo sequence (3D MP-RAGE) anatomical T1-weighted images of the brain, acquired with a 3T Siemens Magnetom Verio Syngo MR B17 at the University Clinic Leipzig. Generalized autocalibrating partially parallel acquisition parallel imaging technique (Griswold et al., 2002; according to the Alzheimer’s Disease Neuroimaging Initiative standard protocol (Wang et al., 2014) was applied using following scanning variables: repetition time/ echo time 2,300 ms/ 2.98 ms; flip angle 9°; slice/ voxel size 1 mm/ 1.0 × 1.0 × 1.0 mm (x × y × z); slices 176; the field of view 256 mm; bandwidth 240 Hz/Px; base resolution 256; scanning time 5 min 10 s. Clinical MRI ratings were performed by neuroradiologists blind to further assessment data. Volumes of intracranial volume (ICV) and total gray and white matter volume derived from FreeSurfer analysis. FreeSurfer (FS) version 6 was used. FS is a free software package developed by the Athinoula A. Martinos Center for Biomedical Imaging of Harvard University^1^. The images were segmented into gray matter maps and matched to a study-specific cerebral-/cerebellar-specific template. For better tissue segmentation and interindividual alignment, the cerebrum and cerebellum were estimated separately. After deleting non-brain tissue, ICV was obtained by adding up the gray matter, white matter, and CSF. Three-dimensional sequences of both hippocampi were reconstructed. Automated segmentation of the hippocampal subfields was performed by an algorithm implemented in FS (Van Leemput et al., 2009). The subfields Cornu Ammonis (CA) 1, CA 2–3, CA 4-Dentate Gyrus, presubiculum, and subiculum were considered for further analysis (Erickson et al., 2011; Brickman et al., 2014). Total (left and right) whole hippocampal and subfield volumes (in mm^3^), as well as ICV, were normally distributed. We adjusted volumes of the whole hippocampus and its subfields for ICV according to (Raz et al., 2005; Kerti et al., 2013) using the following formula: adjusted volume (in mm^3^) = raw volume (in mm^3^) − ß* (ICV − ICVmean) with ß  being the slope of the regression of the respective volume on ICV. Manual quality control of FS labels was done by two experienced staff members individually according to standard operating procedures. Also, a script was programmed for validation of delineation. Analyses were rerun and unusable or inconsistent data were excluded from analyses.

### Mental Demands at Work (MDW)

Mental demands at work (MDW) were investigated as predictors in this study. In standardized interviews, participants provided information on their present or, if retired, on their former occupation. The occupations were translated into English and coded according to the 2010 Standard Occupational Classification of the O*NET database^2^—a validated database containing standardized occupation—specific descriptors that were developed by the US Department of Labor/Employment and Training Administration (USDOL/ETA). The occupation code of every participant came with a great number of variables that describe details of the work tasks. Each variable is indicating on a continuous scale the level (from low to high) on which the person is facing the particular work characteristic. For purpose of analysis, we selected only those variables that describe “mental” demands at work (O*NET variables “Cognitive Abilities” 1.A.1.a–1.A.2.c.3 and “Skills and worker requirements” 2.A.1.a.1–4.A.4.c.3). By following the classification scheme of MDW in previous analyses (Then et al., 2015), relevant variables were combined in clusters of MDW: “language and knowledge,” “information processing,” “mathematics,” “pattern detection,” and “creativity.” The value of each MDW is the mean of the included variables.

### Cognitive Performance

Cognitive performance was used in this study to check whether hippocampal and brain volume mediates the association between mental demands at work and cognitive performance as a precondition for the effect under investigation. Cognitive performance was assessed by trained study assistants and was subject to regular quality control by experienced psychologists. Participants completed the Verbal Fluency Test, the Trail Making Test (TMT), and the Word List Test—subtests of the German version of the neuropsychological test battery of the Consortium to Establish a Registry for Alzheimer’s disease; (CERADplus; Morris et al., 1989). The German version of the CERADplus has been validated (see Aebi et al., 2002). The Verbal Fluency Test is considered to measure verbal abilities, semantic fluency, and semantic memory (Kraan et al., 2013). The participants are instructed to name as many animals as possible in one minute. The participant’s score equals the number of correctly named animals. The TMT is considered to measure working memory, task-switching ability (Salthouse, 2011), and executive control (Arbuthnott and Frank, 2010). The participants are instructed, first, to connect numbers in ascending order as fast as they can (version A) and, second, to connect numbers and letters alternatingly (version B). When an error is made, the participants had to return to the number where the error originated. The participant’s score corresponds to the number of seconds needed to complete the test. The Word List Test is considered to measure memory. The participants are instructed to read out ten words and subsequently recall them. This was repeated three times with the same 10 words. The participant’s score is the number of words remembered correctly.

### Covariates

Age was calculated as the difference in years by subtracting the birth date from the interview date. Gender was estimated using the sex that was recorded in the population registry. Education, as reported by the participant, was categorized as “low” for having completed high school or less, “moderate” for having completed college or a professional training school, and “high” for having completed a university degree. Information on diabetes and hypertension was obtained by asking the participant “Have you ever been diagnosed with …?.” The APOE genotype was identified from peripheral blood leukocytes using an automated protocol on the Qiagen Autopure LS (Qiagen, Hilden, Germany) and by following the method of Aslanidis and Schmitz (1999) *via* Roche Lightcycler 480 (Aslanidis and Schmitz, 1999).

### Statistical Analysis

Statistical analyses were performed using STATA 16 and employed an alpha level for statistical significance of 5% (*p* < 0.05, two-tailed). Bonferroni-correction for the clusters of MDW (five levels) would yield a significance level of *p* < 0.01. All analyses were conducted separately for those actively working and those retired, as associations might be different in people who have retired from their job compared to those who face MDW on a daily level.

Descriptive data analyses on differences in whole hippocampal and brain volume and MDW concerning characteristics of the study sample were conducted *via* analysis of variance (ANOVA) and Pearson’s correlation. The association between hippocampal and brain volume and MDW on cognitive performance was analyzed *via* pairwise comparison correlations. The main research question, the association between MDW and hippocampal and brain volume, was analyzed *via* linear regression analyses, first univariate and then adjusted for factors that might introduce confounding due to known effects on hippocampus volume i.e., gender and age (Pruessner et al., 2001), education (Noble et al., 2012), diabetes (Gold et al., 2007), hypertension (Shih et al., 2016), and APOE e4-allele (Plassman et al., 1997).

The following sensitivity analyses were conducted: as education is an important confounder, we analyzed potential interaction effects using the same linear regression model with an additional interaction term for education and MDW. As hippocampal and brain volume deteriorates with aging, the association under investigation might be age-sensitive. Therefore, we analyzed potential interaction effects using the same linear regression model as before with an additional interaction term for age and MDW [for retired and not-retired individuals together adjusted for being retired (yes/no)]. Finally, we tested whether hippocampal and brain volume mediates the association between MDW and cognitive performance using partial least square structural equation modeling.

## Results

### Hippocampal and Brain Volume

The mean whole hippocampus volume was 7.7 [standard deviation (SD) 0.8], the mean ICV 1,472,315 mm^3^ (SD 147,104.7), the mean gray matter volume 585,876.9 mm^3^ (SD 61,774.2), and the mean white matter 424,326.4 mm^3^ (SD 50,995.6). Associations between personal characteristics and brain parameters are shown in Table 1. Individuals who were retired and those who were older had a smaller hippocampal volume, less ICV, less gray matter, and less white matter (see Tables 1, 2). Individuals with hypertension had a smaller hippocampal volume and less gray matter. There was no significant difference concerning hippocampal volumes and education or APOE e4 allele (see Table 1). A smaller hippocampal volume was associated with poorer performance in the Word List Test and the TMT, and less gray matter was associated with poorer performance in all cognitive tests (see Table 2). In individuals that were not retired, the white matter was associated with performance in the Word List Test and the TMT A (see Table 2).

**Table 1:** Mean hippocampal and brain volume (in mm^3^) concerning the characteristics of the study sample (*n* = 1, 409).

		Hippocampal volume^2^		White matter		Gray matter		ICV	
		Not retired	Retired	Not retired	Retired	Not retired	Retired	Not retired	Retired
	N (%)	721 (47.7)	789 (52.3)	600 (46.3)	697 (53.7)	721 (47.7)	789 (52.3)	721 (47.7)	789 (52.3)
	Mean (SD)	8,110.5 (713.6)	7,343.9 (797.7)	440,912.5 (54,663.3)	410,048.5 (42,772.2)	616,548.2 (61,820.7)	557,849.0 (46,565.5)	1,494,801.0 (148,403.6)	1,451,766.7 (142,937.6)
	F^1^		384.66	129.85		438.9		32.92	
	p^1^		**<0.001**	**<0.001**		**<0.001**		**<0.001**	
**Gender**	Male	8,135.9 (742.9)	7,183.3 (792.4)	463,028.2 (47,883.9)	429.065.3 (41,027.6)	644,587.9 (56,991.8)	580,159.6 (43,521.0)	1,569,507.5 (129,104.9)	1,533,354.7 (121,579.1)
	Female	8,080.0 (676.5)	7,521.1 (766.2)	412,381.6 (49,463.4)	389,616.8 (34,403.8)	582,951.7 (49,438.5)	533,218.2 (36,300.4)	1,405,289.8 (117,332.2)	1,361,693.6 (105,930.0)
	F^1^	1.10	3.89	160.42	187.75	235.6	267.6	314.12	442.97
	p^1^	0.30	**<0.001**	**<0.001**	**<0.001**	**<0.001**	**<0.001**	**<0.001**	**<0.001**
**Education**	Low	8,120.4 (693.1)	7,398.8 (796.8)	437,954.5 (53,649.5)	405,310.3 (43,938.4)	614,713.5 (65,268.6)	550,588.1 (46,902.9)	1,479,338.1 (149,924.9)	1,418,773.8 (143,087.2)
	Moderate	8,193.2 (671.9)	7,337.2 (817.4)	434,644.2 (45,154.7)	404,216.5 (41,484.5)	612,291.5 (60,004.2)	549,241.7 (44,133.9)	1,480,419.5 (135,626.6)	1,433,737.0 (145,244.8)
	High	8,050.2 (753.4)	7,302.5 (785.7)	447,843.9 (60,363.1)	417,585.2 (41,603.8)	620,912.1 (59,457.9)	569,215.9 (45,528.4)	1,518,347.6 (151,877.8)	1,490,322.7 (132,331.5)
	F^1^	2.20	1.07	3.14	7.66	1.23	16.91	5.88	21.48
	p^1^	0.11	0.34	**0.044**	**0.001**	0.293	**<0.001**	**0.003**	**<0.001**
**APOE e4**	No	8,108.5 (715.9)	7,357.3 (805.5)	441,460.1 (52,466.5)	408,982.5 (42,553.7)	616,906.9 (62,950.9)	558,195.9 (46,903.5)	1,494,868.2 (15,271.1)	1,449,386.9 (140,586.4)
	Yes	8,117.2 (707.8)	7,302.1 (773.2)	438,952.2 (62,075.4)	413,485.7 (43,421.4)	615,320.1 (57,952.9)	556,770.2 (45,602.3)	1,494,571.1 (138,582.0)	1,459,166.4 (150,151.5)
	F^1^	0.02	0.70	0.22	1.40	0.08	0.14	0.0	0.68
	P^1^	0.89	0.40	0.643	0.238	0.773	0.712	0.982	0.409
**Diabetes**	No	8,138.2 (700.2)	7,379.1 (789.7)	441,458.6 (55,020.1)	411,409.6 (43,710.5)	617,428.5 (62,295.9)	559,631.1 (47,019.1)	1,494,128.4 (149,160.9)	1,450,186.9 (142,902.6)
	Yes	7,450.3 (723.4)	7,163.7 (816.7)	427,806.8 (44,191.6)	403,160.5 (37,075.9)	595,541.4 (45,017.6)	548,731.3 (43,203.9)	1,510,850.5 (130,274.5)	1,459,849.5 (143,399.9)
	F^1^	26.79	7.94	1.44	3.58	3.50	5.95	0.35	0.49
	p^1^	**<0.001**	**0.005**	0.231	0.059	0.062	**0.015**	0.553	0.483
**Hypertension**	No	8169.2 (699.7)	7426.5 (800.4)	442,415.7 (56,364.8)	411,163.6 (41,694.7)	623,744.7 (62,593.8)	563,508.4 (45,029.6)	1,497,694.8 (145,837.2)	1,449,627.3 (140,500.7)
	Yes	7926.3 (715.9)	7253.4 (793.3)	436,055.2 (49,324.3)	409,596.1 (43,795.8)	599,634.6 (56,702.8)	554,395.7 (48,057.6)	1,484,954.9 (156,973.3)	1,455,907.2 (146,564.7)
	F^1^	14.6	8.42	1.51	0.22	19.01	6.73	0.90	0.34
	p^1^	**<0.001**	**0.004**	0.219	0.638	**<0.001**	**0.009**	0.343	0.561

*Notes: 1, as estimated by analysis of variance (ANOVA); 2, whole hippocampal volume in mm^3^ including left and right hippocampi; ICV, intracranial volume; N, number of participants; p, level of significance; SD, standard deviation; SES, socioeconomic status. Bold indicates significance <0.05*.

**Table 2:** Pearson’s pairwise correlation between hippocampal and brain volume with cognitive performance and mental demands at work (MDW, *n* = 1, 409).

	Hippocampal volume^1^		White matter		Gray matter		ICV	
	Retired	Not retired	Retired	Not retired	Retired	Not retired	Retired	Not retired
	*r* (*p*)	*r* (*p*)	*r* (*p*)	*r* (*p*)	*r* (*p*)	*r* (*p*)	*r* (*p*)	*r* (*p*)
Age	**−0.349 (<0.001)**	**−0.277 (<0.001)**	**−0.174 (<0.001)**	**−0.086 (0.035)**	**−0.138 (<0.001)**	**−0.481 (<0.001)**	0.038 (0.293)	**0.123 (0.001)**
Verbal fluency	0.004 (0.99)	−0.057 (0.12)	0.069 (0.065)	**0.091 (0.026)**	**0.079 (0.028)**	**0.146 (<0.001)**	**0.079 (0.026)**	**0.112 (0.003)**
Word list	**0.199 (<0.001)**	**0.168 (<0.001)**	−0.055 (0.149)	−0.055 (0.181)	**−0.070 (0.048)**	**0.138 (<0.001)**	**−0.168 (<0.001)**	−0.036 (0.330)
Trail making Test A	**−0.154 (<0.001)**	**−0.170 (<0.001)**	−0.074 (0.051)	**−0.129 (0.002)**	**−0.101 (0.005)**	**−0.270 (<0.001)**	0.014 (0.699)	**−0.108 (0.004)**
Trail making Test B	**−0.132 (<0.001)**	**−0.165 (<0.001)**	**−0.085 (0.025)**	−0.077 (0.060)	**−0.108 (0.002)**	**−0.219 (<0.001)**	−0.007 (0.838)	−0.070 (0.059)
MDW language and knowledge	−0.053 (0.14)	0.052 (0.16)	**0.123 (0.001)**	**0.107 (0.009)**	**0.154 (<0.001)**	**0.110 (0.003)**	**0.180 (<0.001)**	**0.087 (0.019)**
MDW information processing	−0.066 (0.06)	0.013 (0.74)	**0.182 (<0.001)**	**0.126 (0.002)**	**0.198 (<0.001)**	**0.123 (0.001)**	** 0.225 (<0.001)**	**0.107 (0.004)**
MDW mathematics	−0.052 (0.15)	0.011 (0.78)	**0.081 (0.034)**	**0.109 (0.007)**	**0.127 (<0.001)**	**0.124 (0.001)**	**0.139 (<0.001)**	**0.118 (0.002)**
MDW pattern detection	−0.035 (0.32)	0.028 (0.45)	**0.158 (<0.001)**	**0.128 (0.002)**	**0.188 (<0.001)**	**0.118 (0.002)**	**0.199 (<0.001)**	**0.095 (0.011)**
MDW creativity	**−0.111 (0.002)**	0.043 (0.25)	**0.153 (<0.001)**	**0.165 (<0.001)**	**0.196 (<0.001)**	**0.158 (<0.001)**	**0.225 (<0.001)**	**0.169 (<0.001)**

*Notes: 1, whole hippocampal volume in mm^3^ including left and right hippocampi; ICV, intracranial volume; p, level of significance; r, Pearson’s correlation coefficient. Bold indicates significance <0.05*.

### Mental Demands at Work (MDW)

The average level of the MDW “language and knowledge” was 3.31 (SD 0.63), of “information processing” was 3.69 (SD 0.80), of “mathematics” was 2.36 (SD 1.06), of “pattern detection” was 2.67 (SD 0.59), and of “creativity” was 2.99 (SD 1.24). Individuals who were retired had, on average, significantly higher levels of “language and knowledge” (3.35 vs. 3.27, *F* = 5.88, *p* = 0.015), “mathematics” (2.49 vs. 2.23, *F* = 22.18, *p* < 0.001), “pattern detection” (2.71 vs. 2.62, *F* = 7.33, *p* = 0.007), and “creativity” (3.09 vs. 2.89, *F* = 9.54, *p* = 0.002), but not of “information processing” (3.73 vs. 3.66, *F* = 3.41, *p* = 0.065). Individuals with higher education had significantly higher MDW levels compared to individuals with lower education (see Table 3). Retired men had significantly higher MDW than women; there was no significant difference in MDW between men and women that were still actively working (see Table 3). Further, there was no significant difference concerning APOE e4 or diabetes status.

**Table 3:** Characteristics of the study sample (*n* = 1,409).

		Retired					Not retired				
		MDW language and knowledge	MDW information processing	MDW mathematics	MDW pattern detection	MDW creativity	MDW language and knowledge	MDW information processing	MDW mathematics	MDW pattern detection	MDW creativity
		M (SD)	M (SD)	M (SD)	M (SD)	M (SD)	M (SD)	M (SD)	M (SD)	M (SD)	M (SD)
**Sex**	male	3.46 (0.67)	3.92 (0.78)	2.67 (1.19)	2.83 (0.59)	3.44 (1.24)	3.25 (0.67)	3.67 (0.81)	2.29 (1.11)	2.65 (0.63)	3.04 (1.26)
	Female	3.24 (0.58)	3.53 (0.80)	2.28 (0.97)	2.56 (0.53)	2.70 (1.19)	3.31 (0.53)	3.65 (0.77)	2.16 (0.84)	2.59 (0.57)	2.72 (1.09)
	F^1^	23.68	47.32	25.08	45.84	72.20	1.85	0.11	2.90	2.04	13.16
	p^1^	**<0.001**	**<0.001**	**<0.001**	**<0.001**	**<0.001**	0.17	0.74	0.09	0.15	**<0.001**
**Education**	low	2.93 (0.49)	3.27 (0.67)	1.96 (0.73)	2.47 (0.45)	2.34 (1.02)	2.97 (0.57)	3.34 (0.78)	1.87 (0.80)	2.43 (0.61)	2.44 (1.08)
	moderate	3.24 (0.61)	3.59 (0.73)	2.34 (1.01)	2.61 (0.55)	2.90 (1.14)	3.24 (0.53)	3.61 (0.65)	2.07 (0.71)	2.62 (0.59)	2.77 (0.93)
	high	3.77 (0.49)	4.21 (0.70)	3.01 (1.19)	2.96 (0.59)	3.82 (1.13)	3.58 (0.55)	3.98 (0.73)	2.67 (1.14)	2.81 (0.56)	3.39 (1.25)
	F^1^	195.39	138.10	83.62	63.81	136.28	80.57	52.06	54.21	29.00	51.23
	p^1^	**<0.001**	**<0.001**	**<0.001**	**<0.001**	**<0.001**	**<0.001**	**<0.001**	**<0.001**	**<0.001**	**<0.001**
**APOE e4**	No	3.36 (0.64)	3.73 (0.81)	2.49 (1.10)	2.72 (0.57)	3.09 (1.27)	3.28 (0.62)	3.65 (0.81)	2.26 (1.03)	2.62 (0.62)	2.89 (1.22)
	Yes	3.32 (0.65)	3.76 (0.83)	2.44 (1.12)	2.67 (0.59)	3.07 (1.28)	3.25 (0.59)	3.68 (0.73)	2.12 (0.87)	2.63 (0.54)	2.87 (1.10)
	F^1^	0.50	0.20	0.41	1.20	0.06	0.29	0.11	2.30	0.02	0.05
	p^1^	0.48	0.66	0.52	0.27	0.81	0.59	0.74	0.13	0.90	0.83
**Diabetes**	No	3.37 (0.64)	3.75 (0.81)	2.50 (1.11)	2.71 (0.59)	3.12 (1.26)	3.28 (0.61)	3.66 (0.79)	2.24 (0.98)	2.63 (0.60)	2.89 (1.18)
	Yes	3.28 (0.63)	3.66 (0.83)	2.39 (1.07)	2.69 (0.55)	2.93 (1.32)	3.18 (0.69)	3.55 (0.88)	1.99 (1.28)	2.53 (0.72)	2.95 (1.60)
	F^1^	1.76	1.35	0.99	0.02	2.51	0.75	0.54	1.67	0.72	0.06
	p^1^	0.18	0.25	0.32	0.89	0.11	0.39	0.47	0.20	0.40	0.81
**Hypertension**	No	2.02 (0.85)	3.74 (0.81)	2.45 (1.09)	2.69 (0.58)	3.06 (1.26)	1.97 (0.79)	3.67 (0.80)	2.23 (0.99)	2.63 (0.60)	2.89 (1.16)
	Yes	2.06 (0.83)	3.73 (0.81)	2.51 (1.13)	2.71 (0.58)	3.11 (1.28)	1.88 (0.79)	3.59 (0.76)	2.19 (1.07)	2.59 (0.63)	2.79 (1.24)
	F^1^	0.38	0.02	0.39	0.22	0.25	1.56	1.19	0.26	0.51	1.00
	p^1^	0.537	0.897	0.533	0.640	0.618	0.213	0.276	0.608	0.475	0.317
		**r (p)^2^**	**r (p)^2^**	**r (p)^2^**	**r (p)^2^**	**r (p)^2^**	**r (p)^2^**	**r (p)^2^**	**r (p)^2^**	**r (p)^2^**	**r (p)^2^**
**Verbal Fluency**		**0.177 (<0.001)**	**0.143 (<0.001)**	**0.118 (0.001)**	0.069 (0.051)	**0.154 (<0.001)**	**0.159 (<0.001)**	**0.120 (0.001)**	0.065 (0.079)	**0.086 (0.021)**	**0.117 (0.002)**
**Wordlist**		**0.102 (0.004)**	0.068 (0.06)	0.050 (0.16)	0.030 (0.40)	0.016 (0.66)	**0.128 (0.001)**	**0.098 (0.009)**	0.069 (0.06)	**0.075 (0.045)**	0.024 (0.53)
**TMT A**		−0.050 (0.16)	−0.040 (0.26)	−0.041 (0.26)	−0.050 (0.16)	−0.030 (0.40)	**−0.107 (0.004)**	**−0.093 (0.013)**	−0.037 (0.33)	**−0.085 (0.023)**	−0.051 (0.17)
**TMT B**		**−0.179 (<0.001)**	**−0.157 (<0.001)**	**−0.165 (<0.001)**	**−0.132 (<0.001)**	**−0.107 (0.003)**	**−0.244 (<0.001)**	**−0.165 (<0.001)**	**−0.146 (<0.001)**	**−0.142 (<0.001)**	**−0.136 (<0.001)**

*Notes: 1, as estimated by analysis of variance (ANOVA); 2, as estimated by pairwise comparison correlations; CI, confidence interval 95%; M, mean; MDW, mental demands at work; N, number of participants, p, level of significance; r, regression coefficient; SD, standard deviation; TMT A, Trail Making Test A with higher scores indicating poorer cognitive performance; TMT B, Trail Making Test B with higher scores indicating poorer cognitive performance. Bold indicates significance <0.05*.

**Figure 1:**
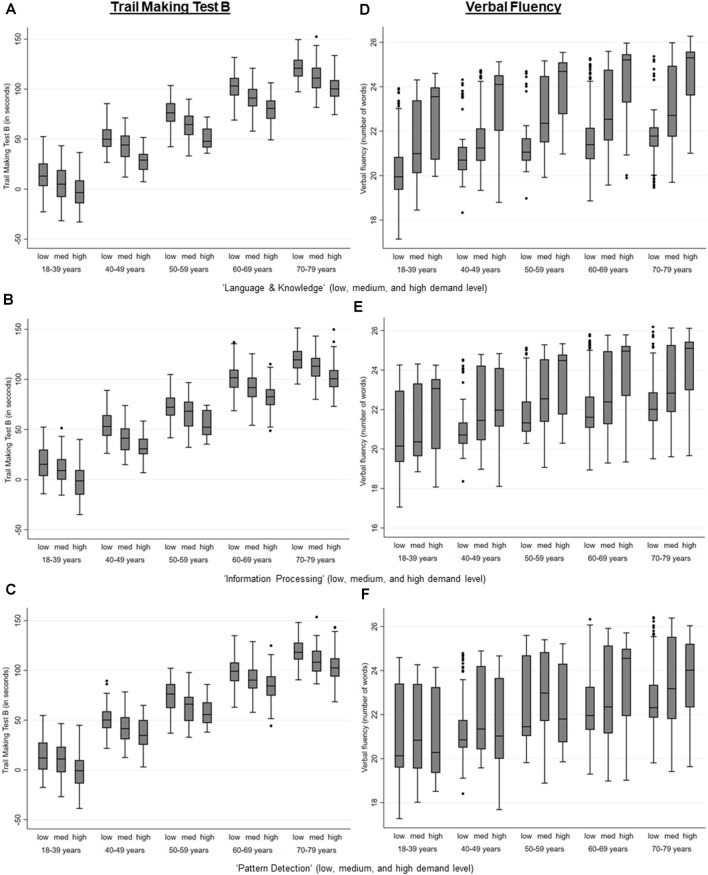
Performance in the Trail Making Test B and the Verbal fluency test by the level of mental demands at work (MDW) and age, estimated *via* regression analyses separate for those that are retired and those that are not retired an adjusted for age, gender, education, APOE e4-allele, hypertension, and diabetes. **(A)** Level of the mental work demand, Language & Knowledge on performance in the Trail Making Test B. **(B)** Level of the mental work demand, Information Processing on performance in the Trail Making Test B. **(C)** Level of the mental work demand, Pattern Detection on performance in the Trail Making Test B. **(D)** Level of the mental work demand, Language & Knowledge on performance in the Verbal Fluency Test. **(E)** Level of the mental work demand, Information Processing on performance in the Verbal Fluency Test. **(F)** Level of the mental workdemand, Pattern Detection on performance in the Verbal Fluency Test.

Higher MDW were significantly associated with a better performance in cognitive tests that measured executive cognitive abilities (all *p* < 0.001; TMT B, Verbal Fluency Test), whilst the performance in the TMT A and the Word List Test was only associated with a higher level in the MDW “language and knowledge,” “information processing,” and “pattern detection” among those who were not yet retired (see Table 3, see also Figure 1).

### Association of Demands With Hippocampal and Brain Volume

**Figure 2:**
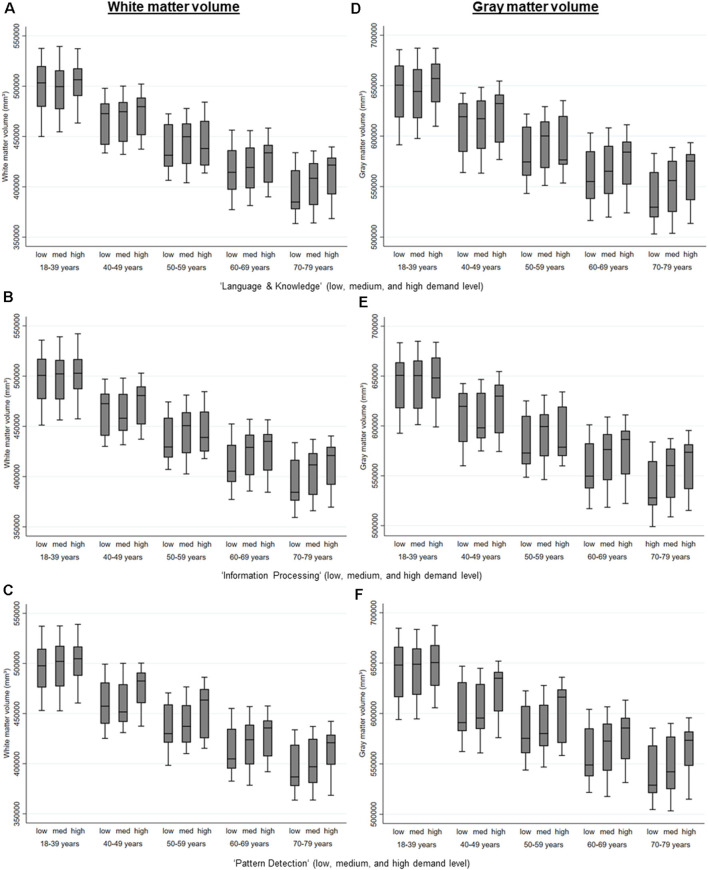
White matter and gray matter volume by the level of MDW and age, estimated *via* regression analyses separate for those that are retired and those that are not retired and adjusted for age, gender, education, APOE e4-allele, hypertension, and diabetes. **(A)** Level of the mental work demand, Language & Knowledge on white matter volume. **(B)** Level of the mental work demand, Information Processing on white matter volume. **(C)** Level of the mental work demand, Pattern Detection on white matter volume. **(D)** Level of the mental work demand, Language & Knowledge on gray matter volume. **(E)** Level of the mental work demand, Information Processing on gray matter volume. **(F)** Level of the mental work demand, Pattern Detection on gray matter volume.

Higher MDW was associated with more white and gray matter (see Table 2 and Figure 2). If adjusted for confounders, only higher MDW “information processing” was associated with larger white and gray matter volume, higher MDW “creativity” and “language and knowledge” only if not yet retired (see Table 4). Higher MDW “pattern detection” was only significantly associated with more white and gray matter volume if the persons had retired (see Table 4).

**Table 4:** Results of the regression analyses on the association between MDW and hippocampal and brain volume, separate for those that are retired and those that are not retired, adjusted for age, gender, education, APOE e4-allele, hypertension, and diabetes.

	Unadjusted				Adjusted^1^			
	Retired		Not retired		Retired^1^		Not retired^1^	
Hippocampal volume^2^								
	*b* (CI 95%)	*P*	*b* (CI 95%)	*P*	*b* (CI 95%)	*P*	*b* (CI 95%)	*P*
MDW language and knowledge	−65.5 (−156.5; 25.6)	0.16	61.1 (−26.9; 148.9)	0.17	−16.2 (−91.1; 58.7)	0.671	59.4 (−10.9; 129.7)	0.098
MDW information processing	−64.9 (−136.3; 6.3)	0.07	11.3 (−54.9; 77.6)	0.74	−10.1 (−86.5; 66.3)	0.795	−10.7 (−82.6; 61.2)	0.771
MDW mathematics	−37.5 (−88.9; 13.9)	0.15	7.5 (−48.7; 63.8)	0.79	1.6 (−51.1; 54.3)	0.952	−4.1 (−63.4; 55.3)	0.893
MDW pattern detection	−48.5 (−145.7; 48.6)	0.33	33.4 (−55.4; 122.3)	0.46	43.5 (−53.5; 140.5)	0.379	1.4 (−90.9; 93.7)	0.976
MDW creativity	−69.9 (−114.9; -24.9)	**0.002**	25.5 (−21.6; 72.6)	0.29	−21.1 (−69.8; 27.7)	0.396	34.4 (−13.9; 82.7)	0.162
**Hippocampal subfields^2,3^ CA2&3**								
MDW pattern detection	123.3 (22.5; 224.2)	**0.017**	17.6 (−87.5; 122.8)	0.742	131.1 (22.9; 239.4)	**0.018**	−35.9 (−143.8; 72.1)	0.514
**White matter volume**								
MDW language and knowledge	8,242.8 (3,495.2; 12,990.4)	**0.001**	9,563.9 (2,126.8; 17,000.9)	**0.012**	3,773.7 (−323.7; 7,871.0)	0.071	8,356.7 (3,285.4; 13,428.1)	**0.001**
MDW information processing	9,657.0 (5,903.1; 13,410.9)	**<0.001**	8,781.2 (2,864.1; 14,698.4)	**0.004**	5,089.9 (983.9; 9,195.8)	**0.015**	5,680.8 (106.3; 11,255.3)	**0.046**
MDW mathematics	3,133.7 (410.6; 5,856.7)	**0.024**	6,004.3 (1,982.5; 10,026.0)	**0.003**	628.5 (−2,187.6; 3,444.5)	0.661	1,915.3 (−2,294.8; 6,125.5)	0.372
MDW pattern detection	11,809.8 6,388.5; 17,231.2)	**<0.001**	11,556.2 (4,108.8; 19,003.7)	**0.002**	5,491.9 (72.6; 10911.4)	**0.047**	5,923.1 (−1005.9; 12852.1)	0.094
MDW creativity	5,142.6 (2,787.8; 7,497.4	**<0.001**	7,603.6 (4,137.4; 11,069.8)	**<0.001**	1,694.5 (−925.4; 4,314.2)	0.205	3,827.9 (527.7; 7,128.1)	**0.023**
**Gray matter volume**								
MDW language and knowledge	11,172.7 (6,316.2; 16,029.3)	**<0.001**	11,125.8 (3,879.4; 18,372.2)	**0.003**	5,435.6 (1,252.9; 9,618.4)	**0.011**	6,654.2 (2,033.8; 11,274.7)	**0.005**
MDW information processing	11,376.7 (7,482.1; 15,271.4)	**<0.001**	9,549.5 (3,637.8; 15,461.2)	**0.002**	4,847.6 (508.1; 9,187.2)	**0.029**	4,728.1 (14.1; 9,442.1)	**0.049**
MDW mathematics	5,328.8 (2,706.5; 7,951.2)	**<0.001**	7,697.3 (3,159.7; 12,234.9)	**0.001**	1,821.6 (−956.4; 4,599.5)	0.198	3,222.3 (−522.4; 6,967.1)	0.092
MDW pattern detection	15,126.9 (9,957.7; 20,296.2)	**<0.001**	12,025.1 (4,776.6; 19,273.6)	**0.001**	6029.2 (732.8; 11,325.6)	**0.026**	4,171.3 (−1,699.7; 10,042.3)	0.163
MDW creativity	7,175.6 (4,732.4; 9,618.8)	**<0.001**	8,178.6 (4,126.6; 12,330.6)	**<0.001**	2,294.5 (−432.3; 5,021.4)	0.099	3,895.6 (657.0; 7,134.2)	**0.018**
**ICV**								
MDW language and knowledge	40,294.2 (25,402.5; 55,185.9)	**<0.001**	21,223.4 (3,182.7; 39,264.1)	**0.021**	12,026.3 (−330.9; 24,383.4)	0.056	5,435.6 (1,252.9; 9,618.4)	**0.011**
MDW information processing	39,658.5 (27,516.9; 51,800.2)	**<0.001**	20,100.5 (5,650.9; 34,550.0)	**0.006**	13,828.0 (790.4; 26,865.6)	**0.038**	10,287.7 (−2,802.9; 23,378.3)	0.123
MDW mathematics	17,967.4 (9,707.6; 26,227.4)	**<0.001**	17,513.9 (6,801.5; 28,226.4)	**0.001**	2,385.2 (−5994.1; 10,764.5)	0.576	6,324.8 (−4,400.9; 17,050.6)	0.247
MDW pattern detection	49,191.9 (32,572.5; 65811.2)	**<0.001**	23,164.2 (5,368.9; 40,959.4)	**0.011**	11287.2 (−5,167.2; 27,741.5)	0.178	5,461.1 (−11,188.2; 22,110.4)	0.520
MDW creativity	25,298.9 (17,895.6; 32,702.3)	**<0.001**	21,021.4 (11,754.3; 30,288.4)	**<0.001**	4,554.3 (−3,702.4; 12,811.1)	0.279	6,743.5 (−1,869.7; 15,368.8)	0.125

*Notes: 1, model adjusted for age, gender, education; 2, whole hippocampal volume in mm^3^ including left and right hippocampi; 3, significant findings only; APOE e4-allele, hypertension, and diabetes; b, regression coefficient; CA, cornu ammonis; CI, confidence interval 95%; ICV, intracranial volume; MDW, mental demands at work; P, level of significance. Bold indicates significance <0.05*.

For hippocampal volume, there was only one significant association between hippocampal volume and MDW in the descriptive univariate analyses: Among those retired, a higher level of “creativity” correlated with a smaller hippocampal volume (see Table 2). This association was no longer present when accounting for confounders (see Table 4). We considered the different hippocampal subfields and observed that a higher level of the MDW “pattern detection” was associated with larger hippocampal subfields CA2/CA3 in retired individuals (see Table 4). The estimates point out that the difference between those with high and low demands is greater in younger age and decreases with older age.

### Sensitivity Analyses

Interaction effects between education and MDW were found for retired individuals. Education interacts with “pattern detection” on white matter volume (moderate education *b* = −19,831.3, CI95 −34,962.9 to −4,699.8, *p* = 0.010; high education *b* = −13,588.1, CI95 −27,207.3 to −31.1, *p* = 0.051) and intracranial volume (moderate education *b* = −49,895.7, CI95 −97,128.5 to −2,662.9, *p* = 0.038), indicating that individuals with low education have a greater increase in white matter and intracranial volume if they work in jobs with higher MDW “pattern detection” than individuals with moderate education. For individuals who were not retired, education interacted with “creativity” on white matter volume (moderate education *b* = 11749.9, CI95 2982.3–20517.6, *p* = 0.009), indicating that low-educated individuals have a greater increase in the white matter if they work in jobs with higher MDW “creativity” than moderately-educated individuals.

Interaction with age was found for higher MDW “creativity” (*b* = −211.6, CI95 −352.9 to −70.2, *p* = 0.003) and “language and knowledge” (*b* = −271.9, CI95 −500.5 to −43.5, *p* = 0.020) on white matter. Higher MDW “creativity” interacted also with age on total hippocampal volume (*b* = −3.4, CI95 −5.5 to −1.4, *p* = 0.001), indicating that hippocampal volume declines faster in the age period 40–60 in individuals with higher MDW “creativity” than in individuals with low demands.

The potential mediating effects of hippocampal and brain volume on the association of HDW on cognitive performance were analyzed *via* structural equation modeling using latent factor variables. Results are shown in Table 5. For retired individuals, higher MDW was significantly associated with better performance in cognitive testing. For individuals not yet retired, higher MDW was significantly associated with better performance in cognitive testing as well as hippocampal and brain volume. For both groups, there were no indirect associations from MDW to cognitive performance *via* hippocampal and brain volume. Covariates were significantly associated with hippocampal and brain volume and, in this way, also indirectly associated with cognitive performance.

**Table 5:** Results of the structural equation modeling on the association of MDW and hippocampal and brain volume (HBV) on cognition (COG).

	Retired				Not retired			
	Loading	*P*			Loading	*P*		
**Mental demands at work (MDW)**								
MDW language and knowledge	0.898	<0.001			0.891	<0.001		
MDW information processing	0.908	<0.001			0.912	<0.001		
MDW mathematics	0.873	<0.001			0.840	<0.001		
MDW pattern detection	0.826	<0.001			0.844	<0.001		
MDW creativity	0.886	<0.001			0.867	<0.001		
**Hippocampal and brain volume (HBV)**^1^								
Hippocampal volume	−0.085	0.026			0.460	<0.001		
White matter	0.900	<0.001			0.821	<0.001		
Gray matter	0.922	<0.001			0.944	<0.001		
Intracranial volume	0.959	<0.001			0.862	<0.001		
**Cognition (COG)**								
TMT B	0.776	<0.001			0.799	<0.001		
TMT A	0.539	<0.001			0.785	<0.001		
Verbal fluency	0.795	<0.001			0.514	<0.001		
Word list	0.208	<0.001			0.635	<0.001		
**Covariates**								
Education	0.562	<0.001			0.031	0.447		
Age	0.037	0.336			0.860	<0.001		
Gender	0.923	<0.001			0.344	<0.001		
APOE e4	0.045	0.236			0.147	<0.001		
Diabetes	0.085	0.026			0.341	<0.001		
Hypertension	0.059	0.121			0.552	<0.001		
	*b*	*P*	Indirect (*b*)	Total (*b*)	*b*	*p*	Indirect (*b*)	Total (*b*)
**MDW > HBV**	0.001	0.988			0.109	0.002		
**MDW > COG**	0.173	<0.001	0.000	0.173	0.100	0.008	−0.000	0.100
**HBV > COG**	0.080	0.082			−0.001	0.989		
**Covariates > HBV**	−0.580	<0.001			−0.503	<0.001		
**Covariates > COG**	−0.031	0.523	−0.046	−0.077	−0.424	<0.001	0.000	−0.423

*Notes: 1, whole hippocampal volume in mm^3^ including left and right hippocampi; b, regression coefficient; CA, cornu ammonis; CI, confidence interval 95%; ICV, intracranial volume; MDW, mental demands at work; P, level of significance; SE, standard error; TMT, trail making test*.

## Discussion

Since previous studies have shown that high levels of mental demands at work (MDW) are associated with lower dementia risk (Then et al., 2015), this study aimed to investigate whether higher MDW protects cognitive health by preserving hippocampal volume. Findings indicate only a significant association between higher MDW “pattern detection” (not any other MDW) with larger hippocampal subfields CA2 / CA3 in retired individuals. However, our sample comprised only community-based individuals without dementia and we cannot derive any conclusions on individuals with severe cognitive impairments. It is conceivable that associations between MDW and hippocampal volume are sensitive to the neurodegenerative processes of dementia, studies using clinical samples may observe stronger effects on hippocampal size.

The lack of a general association between MDW and hippocampal volume in our sample could be explained by multiple factors. On the one hand, a (neuro)protective effect of MDW may not be related to higher hippocampal plasticity. A study that investigated associations between occupational complexity and hippocampal volume in Alzheimer patients found no association (Boots et al., 2015). Then again, crude hippocampus volume might not be a valid biomarker for hippocampal plasticity. Besides volume, hippocampus microstructure and functional connectivity measured using diffusion-weighted imaging and resting-state BOLD-fMRI has been implicated in cognitive functions and might be a more sensitive marker of cognitive impairment or dementia (Zhou et al., 2008; Fellgiebel and Yakushev, 2011). For example, the functional coupling of the hippocampus with dorsal sub-regions is essential for learning (Mattfeld and Stark, 2015) and mental demands at work that would strengthen functional connectivity of the hippocampus could therefore help to maintain learning abilities longer in life. As cognitive training has shown to promote hippocampal functioning (Rosen et al., 2011; Kirchhoff et al., 2012), likely, working longer in a mentally stimulating job could at least maintain hippocampal functionality for a longer life period.

Findings from our study emphasize that higher MDW, especially in “language and knowledge,” “information processing,” and “creativity,” are associated with better performance in cognitive tests. This confirms a possible protective association between higher levels of those MDW and better cognitive health in old age, as a previous study has shown concerning dementia risk (Then et al., 2015). For those who were retired, only the association with executive cognitive abilities (Trail Making Test B, Verbal Fluency) retained significance, indicating that the effect might be stronger during the active workforce participation. Indeed, previous studies have shown that older age of retirement was associated with a reduced dementia risk (Dufouil et al., 2014) and that high complexity at work seems to facilitate cognitive functioning before retirement (Finkel et al., 2009). This observation is in line with the “use-it-or-lose-it” theory, which states that the active use of cognitive abilities prevents their deterioration in old age (Salthouse, 2006). Nonetheless, there is always the possibility of reverse causality so that individuals with more resilience to cognitive decline work in jobs with higher demands. Even though a great number of previous studies demonstrated that the training of cognitive abilities delays cognitive decline (Rizkalla, 2018). Irrespective of whether innate resilience or training effects establish a protective association between work demands and cognitive health in old age, it seems that the use of semantic long-term memory involved in complex information processing tasks plays a major role. There is no research so far explaining this observation.

As “language and knowledge” and “information processing,” identified as possibly protective MDW in a previous study (Then et al., 2015), were both associated with gray matter in our analysis, it is conceivable that their effect on cognitive performance might be mediated through their effect on gray matter. In previous studies, cognitive training has been shown to lead to changes in gray matter (Ceccarelli et al., 2009; Kühn et al., 2014), which could counteract the deterioration of gray matter in aging (Alexander et al., 2006) and Alzheimer’s disease (Karas et al., 2004). However, in our study, there was no indirect path to cognitive functioning. Accordingly, there seem to be independent associations between higher demands and brain volume and higher demands and cognitive performance as well as between brain volume and cognitive performance. A lack of association between brain and cognitive functioning in individuals with high intellectual stimulation during their life-course is commonly referred to as cognitive reserve (Stern, 2002) and is a well-known phenomenon. Hence, it is likely that intellectual stimulation at work, especially in the MDW “language and knowledge,” “information processing,” and “creativity,” take a cognitive reserve-effect, in addition to effects that they have on the brain.

While it is intuitive to see high demands involving language and knowledge and more complex information processing as cognitive training at work, this understanding does not come so obviously for creativity. In our findings, there were associations between higher MDW “creativity” and more gray matter as well as white matter volume among individuals that were still working. It also seems that low-educated individuals can benefit from the effects of MDW “creativity.” There is not yet any consensus on how creative activity shows in or alters the brain (Arden et al., 2010). It might be domain-specific (Boccia et al., 2015); norepinephrine (Heilman, 2016) and the functional connectivity of the default mode network might play a role (Beaty et al., 2014). Thus, more research is necessary to understand potential effects.

Our study is not without limitations. First, it is only cross-sectional. Longitudinal analyses would reveal changes in hippocampal volume in each individual over time—this would give more information on a possible causal pathway. Moreover, we did not include clinical dementia cases. Our study might therefore underestimate the true effect size in the general elderly population. Further, even though we adjusted for important socioeconomic and lifestyle factors, there might be others affecting the results, which we did not consider. Therefore, novel model prediction statistics that are better capable to account for in-depths individual differences, collinearities, and multiple data points might be used in future analyses of large MRI datasets (Bzdok and Yeo, 2017). Finally, 1 mm^3^ isotropic data lacks sufficient resolution and contrast for the visualization of the internal structure of the hippocampus (Elman et al., 2019; Wisse et al., 2020) so that the estimates for hippocampal subfields may not be as optimal as desired. Further studies are necessary to confirm our findings for the subfields. Strengths of our study include the large sample size, the well-defined population under study, and the state-of-the-art 3T neuroimaging protocol.

## Conclusions

In trying to gain a better understanding of how mentally demanding activities at work (MDW) influence dementia risk, our analysis explored whether high demands could maintain or even enhance the plasticity of the hippocampus. Our findings do not support the notion that MDW protects cognitive health *via* hippocampal volume, neither *via* brain volume. Yet, we observed associations between higher demands in “language and knowledge,” “information processing,” and “creativity” at work on larger white and gray matter volume and better cognitive functioning. “Creativity,” however, seems to be more relevant for individuals that are still actively working; the protective effect might disappear when the person retires. Among retired individuals, on the other hand, higher demands in “pattern detection” were associated with larger white matter volume as well as larger hippocampal subfields CA2/CA3, suggesting a retention effect later in life. This finding is so important because a previous study has identified this MDW as well as the other three previously mentioned MDWs to be associated with a lower dementia risk (Then et al., 2015). At this point, it is unclear what the biological pathways are connecting them to cognitive health in old age. Further studies are necessary to evaluate how individuals who have high levels of mental demands at work gain resilience against dementia.

## Data Availability Statement

The datasets of the “Adult Study” of the Leipzig Research Centre for Civilization Diseases (LIFE) are available upon request from the authors. Requests to access the datasets should be directed to Christoph Engel, christoph.engel@imise.uni-leipzig.de.

## Ethics Statement

The studies involving human participants were reviewed and approved by Ethics Committee of the Medical Faculty of the University of Leipzig. The patients/participants provided their written informed consent to participate in this study.

## Author Contributions

CE, ML, JT, AV, and SR-H contributed to the conception and design of the study. FR, SH, MS, CE, RBa, RBu, TL, and AW contributed to the data collection, data preparation, and data quality control. FR performed the statistical analysis and wrote the first draft. SH, WV, MA, and AW interpreted the data and/or wrote sections of the draft. All authors contributed to the article and approved the submitted version.

## Conflict of Interest

The authors declare that the research was conducted in the absence of any commercial or financial relationships that could be construed as a potential conflict of interest.

## Footnotes

^1^https://surfer.nmr.mgh.harvard.edu/

^2^http://www.onetonline.org
